# Empirical evaluation of data normalization methods for molecular classification

**DOI:** 10.7717/peerj.4584

**Published:** 2018-04-11

**Authors:** Huei-Chung Huang, Li-Xuan Qin

**Affiliations:** Department of Epidemiology and Biostatistics, Memorial Sloan Kettering Cancer Center, New York, NY, USA

**Keywords:** Microarray, Normalization, Classification, Validation

## Abstract

**Background:**

Data artifacts due to variations in experimental handling are ubiquitous in microarray studies, and they can lead to biased and irreproducible findings. A popular approach to correct for such artifacts is through post hoc data adjustment such as data normalization. Statistical methods for data normalization have been developed and evaluated primarily for the discovery of individual molecular biomarkers. Their performance has rarely been studied for the development of multi-marker molecular classifiers—an increasingly important application of microarrays in the era of personalized medicine.

**Methods:**

In this study, we set out to evaluate the performance of three commonly used methods for data normalization in the context of molecular classification, using extensive simulations based on re-sampling from a unique pair of microRNA microarray datasets for the same set of samples. The data and code for our simulations are freely available as R packages at GitHub.

**Results:**

In the presence of confounding handling effects, all three normalization methods tended to improve the accuracy of the classifier when evaluated in an independent test data. The level of improvement and the relative performance among the normalization methods depended on the relative level of molecular signal, the distributional pattern of handling effects (e.g., location shift vs scale change), and the statistical method used for building the classifier. In addition, cross-validation was associated with biased estimation of classification accuracy in the over-optimistic direction for all three normalization methods.

**Conclusion:**

Normalization may improve the accuracy of molecular classification for data with confounding handling effects; however, it cannot circumvent the over-optimistic findings associated with cross-validation for assessing classification accuracy.

## Background

Microarray data are prone to data artifacts that arise from variations in experimental handling factors such as the technician and the processing batch (Ransohoff, 2005; Baggerly, Coombes & Neeley, 2008; Leek et al., 2010). Statistical methods for post hoc data normalization have been developed to remove such data artifacts and improve the accuracy and reproducibility of data inferences (Huber et al., 2002; Yang et al., 2002; Bolstad et al., 2003; Irizarry et al., 2003). The performance of normalization methods has been extensively studied, focusing primarily on the discovery of molecular biomarkers that are differentially expressed between sample groups (Wu et al., 2004; Johnstone et al., 2013; Qin & Zhou, 2014). Very few studies have been reported on their performance for the development of a multi-marker classifier, an increasingly important application of microarray data in the era of precision medicine (Simon et al., 2003; Simon, 2005; McShane et al., 2013).

We set out to investigate the performance of normalization methods for molecular classification, using a unique pair of microarray datasets that we previously collected (Qin et al., 2014). The same set of tumor samples were profiled twice using microRNA microarrays, *once* with uniform handling and blocked randomization when allocating arrays to samples and *a second time* with non-uniform handling and arrays allocated in the order of sample collection. Throughout the paper, these two datasets are referred to as the *uniformly handled dataset* and the *non-uniformly handled dataset*, respectively. The uniformly handled data possessed minimal handling effects, so it was used to approximate the biological effects for each tumor sample; the non-uniformly handled data exhibited obvious handling effects and, for each array in this dataset, its handling effects were estimated as its difference from the matched array in the uniformly handled dataset. Two-thirds of the samples were randomly selected and two-thirds of the array were non-randomly selected to simulate the *training data*, through a process that we call “virtual re-hybridization” (i.e., summing the biological effects of a sample with the handling effects of its assigned array) (Qin et al., 2014). Biological effects for the remaining third of the samples were used to serve as the *test data*. A classifier was built using the training data and its accuracy was assessed by external validation using the test data. We have previously shown that cross-validation is prone to biased estimation of prediction accuracy when handling effects are pronounced in the data being analyzed, despite of the use of quantile normalization (Qin, Huang & Begg, 2016); therefore, we used external validation as the primary approach for assessing classification accuracy when evaluating the impact of normalization methods.

We examined the use of three normalization methods (median normalization, quantile normalization, and variance stabilizing normalization), in comparison with no normalization, for the training data (Huber et al., 2002; Yang et al., 2002; Bolstad et al., 2003; Irizarry et al., 2003). We found that their level of benefits and relative order of performance depend on the level of molecular signal strength, the amount and distributional characteristics of confounding handling effects, and the choice of classification method. We further assessed the use of cross-validation for accuracy assessment following data normalization using median normalization or variance stabilizing normalization. The three normalization methods examined in this paper were chosen based on their popularity in the literature and representation of three families of normalization methods based on global scaling, regression adjustment, and data transformation, respectively. We have made the data and code used in our paper publicly available so that other researchers can explore this topic further.

## Methods

### Data collection

A total of 96 endometrioid endometrial cancer and 96 serous ovarian cancer samples were used in our study. The tumors were all primary, untreated, and collected at Memorial Sloan Kettering Cancer Center between 2000 and 2012. Their use in our study was approved by the Memorial Sloan Kettering Cancer Center Institutional Review Board (Approval No. 12-064).

Two microRNA microarray datasets, one uniformly handled and the other non-uniformly handled, were generated for the 192 samples, using the Agilent Human microRNA microarray (Release 16.0 Agilent Technologies, Santa Clara, CA, USA), which contains 3,523 markers representing 1,347 miRNAs.

When generating the uniformly handled dataset, arrays were allocated to samples using blocked randomization and all arrays were handled by one technician in one processing run. Agilent microRNA arrays come with eight arrays on each array slide, arranged as two rows by four columns, so each array slide served as a “block” of experimental units. In order to avoid any positional effect on the slide, arrays were further stratified by slide row and column, with equal numbers of arrays assigned to the two groups on each row and each column. For a two-by-four array slide, there are a total of six possible configurations that allow row and column balance. When implementing the array assignment for the uniformly handled data, first, 192 arrays from 24 array slides were used for the 192 tumor samples; second, the 24 slides were randomly assigned to four repetitions of the six row–column-balanced configurations; third, arrays randomly assigned to a tumor group as the result of the second step were then randomly allocated to samples in that group.

The non-uniformly handled dataset was processed by two technicians in multiple batches, with arrays assigned to samples in the order of sample collection. This design deliberately imposed non-uniform handling in this dataset to mimic typical practice.

This pair of datasets was used to simulate data to examine the performance of data normalization for the problem of molecular classification, where sample group (endometrial vs ovarian cancer) was the outcome variable of interest. Further details on data collection can be found in our previous publication (Qin et al., 2014).

### Data simulation

Among the 3,523 markers on the Agilent arrays, 351 were significantly differentially expressed (*p*-value < 0.01) between the two tumor groups (in the uniformly handled dataset). To mimic a typical level of molecular signal in a molecular classification study, we reduced the size of between-group-differences for each differentially expressed biomarker by half, resulting in only 63 differentially expressed markers (Qin, Huang & Begg, 2016). In this paper, we report the results for the reduced signal if not stated otherwise. The biological effects of a sample were estimated as its uniformly handled data. The handling effects of an array from the non-uniformly handled dataset were estimated by the difference between its data and the data of its matched array (for the same tumor sample) in the uniformly handled dataset.

In classification studies, a classifier is built on a training set and its classification accuracy evaluated either through cross-validation (i.e., rotational validation based on random splits of the training set) or external validation (using an independent test set). To generate a training set (*n* = 64 + 64) and an independent test set (*n* = 32 + 32) for the purpose of external validation, we split the samples *randomly* into such two sets and the arrays *non-randomly* with the first 64 arrays (handled by one technician in two batches) and the last 64 arrays (handled by another technician in two batches) assigned to the training set (Fig. S1). This split of arrays ensured that handling effects of both technicians are sufficiently represented in the training set.

To generate the training data under the scenario where array handling confounded with the sample group, we assigned 90% of the first 64 arrays and 10% of the last 64 arrays to endometrial samples, and the rest of the arrays to ovarian samples. We then virtually re-hybridized each sample to its assigned array by summing the biological effects of a sample to the handling effects of its assigned array. One hundred training datasets were generated for each simulation scenario. For the test data, we used only the biological effects for the test set samples to mimic an unambiguously ideal scenario.

Handling effects in our empirical data arose from the use of multiple array processing technicians and multiple processing batches in the same laboratory facility. To mimic a more heterogeneous level of handling effects when the data had been collected in multiple laboratories, we considered two methods to amplify the level of handling effects: (1) adding a constant of 1/2 to the handling effects of half of the arrays; (2) scaling handling effects to the power of different constants (1/3, 2/3, 1.2, 1.3) for arrays in the four batches. These two studies are referred to as amplified handling effects by location shift and by scale change, respectively. In practice, location shift in array data can result from, for example, unequal quantities of starting RNA, and scale change can result from, for example, different labeling or detection efficiencies following non-uniform handling of the sample and array processing (Quackenbush, 2002).

### Data preprocessing

Training data and test data were each preprocessed using log2 transformation, data normalization, and then median summarization (across replicate probes for each marker). Detailed justifications on the method choices for the preprocessing steps can be found in our previous publications using this pair of datasets (Qin et al., 2014; Qin, Huang & Zhou, 2014; Qin & Zhou, 2014).

Methods for training data normalization included median normalization, quantile normalization using the {preprocessCore} package (Bolstad et al., 2003; Bolstad, 2016), variance stabilizing normalization using the {vsn} package (Huber et al., 2002), as well as no normalization for comparison.

Test data were “frozen” normalized to training data (McCall, Bolstad & Irizarry, 2010; Qin, Huang & Begg, 2016). That is, the summary distribution of the normalized training data was saved as the reference distribution, and then used to normalize the test data. More specifically, frozen median normalization is to shift the test data to share the same median as the median-normalized training data; frozen quantile normalization adjusts the test data to have the same quantiles as the quantile-normalized training data; frozen variance stabilizing normalization transforms the test data using the parametric transformation estimated in the training data.

### Classification analysis

We used two classification methods in our study: a non-parametric method (PAM) (Tibshirani et al., 2002) and a parametric method (LASSO) (Tibshirani, 1996), using the {pamr} package (Hastie et al., 2014) and the {glmnet} package (Friedman, Hastie & Tibshirani, 2010), respectively. Both methods require the specification of tuning parameters, which were chosen based on five-fold cross-validation. In particular, for LASSO, we followed the recommendation of {glmnet} by selecting the lambda that is one standard error smaller than the lambda that has the smallest misclassification error in order to maintain a more parsimonious model. Tuning parameter selection and feature selection were both done within each iteration of cross-validation (using four folds of the training data). After a classifier was built in the training data, its accuracy was assessed using both external validation in the test data and five-fold cross-validation in the training data, measured as the misclassification error rate (i.e., the proportion of misclassified samples).

### Data and code availability

All analyses were performed using *R 3.3.0*. The paired datasets and R code used in our simulation are available as R packages for free download at GitHub: PRECISION.DATA (https://github.com/LXQin/precisiondata) and PRECISION (https://github.com/LXQin/precision). The raw data for the paired datasets can also be accessed at Gene Expression Omnibus through a SuperSeries record (GSE109059).

## Results

### Comparison of normalization methods in the presence of confounding handling effects

**Figure 1:**
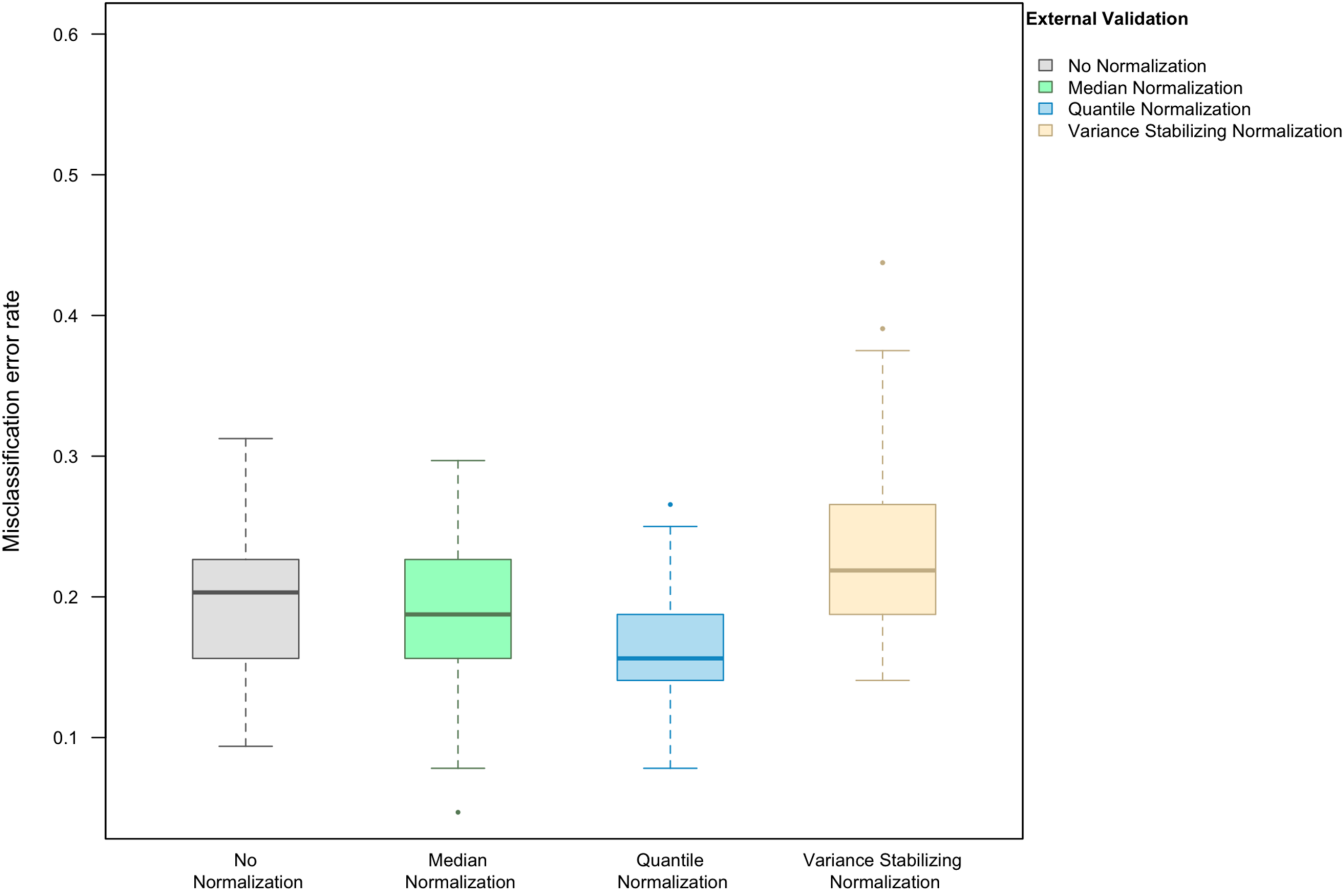
Boxplot of the misclassification error rate based on external validation when using the PAM method for building a classifier. The simulated training data possessed no confounding handling effects. *x*-axis indicates the normalization method for the training data; *y*-axis indicates the misclassification error rate as a percentage.

Here we focus on the results of the simulation study using PAM as the classification method and external validation for assessing the misclassification error rate. We observed that, in the absence of handling effects (i.e., when only using biological effects for samples in the training set as the training data), the median (IQR = inter-quartile range) of the misclassification error was (20.3%, IQR 15.6–22.3%) (Fig. 1).

**Figure 2:**
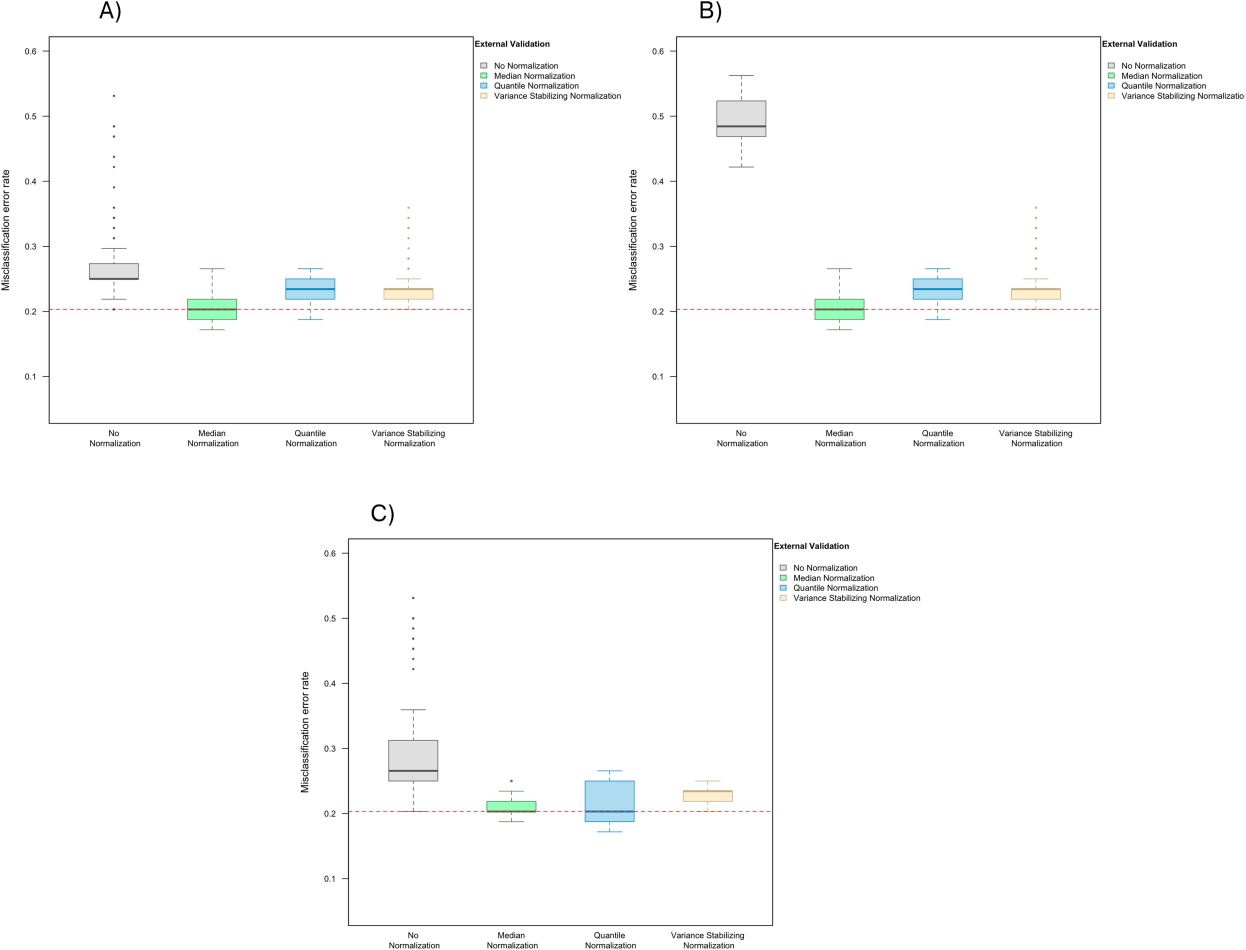
Boxplot of the misclassification error rates based on external validation when using the PAM method for building a classifier. The simulated training data possessed confounding handling effects. *x*-axis indicates the normalization method for the training data; *y*-axis indicates the misclassification error rate as a percentage. (A–C) display the misclassification error when handling effects in the simulated training data were not amplified (A), amplified by location shift (B), and amplified by scale change (C). In (A–C) the horizontal red line indicates the level of classification error rate in the ideal case when the training data possessed no confounding handling effects and received no normalization.

In the presence of handling effects in the training data, the median error rate increased noticeably to 25.0% (IQR 25.0–27.0%) before the use of normalization (Fig. 2A) Data normalization decreased the error rate to 20.3% (IQR 18.8–21.9%) for median normalization, 23.4% (IQR 21.9–25.0%) for quantile normalization, and 23.4% (IQR 21.9–23.4%) for variance stabilizing normalization (Fig. 2A). Among the three methods, median normalization slightly outperformed the other two normalization methods. Moreover, median normalization and quantile normalization were associated with fewer outlying error rates across the simulation runs.

In addition to using a matching normalization method for the test data (for example, “frozen median normalization” was used for test data when “median normalization” was used for training data), we also repeated the analysis comparing training set normalization methods when using only frozen quantile normalization for the test data, and observed similar results (Fig. S2).

### Effect of normalization depends on the level of signal-to-noise ratio and the distributional characteristics of confounding handling effects

When handling effects were amplified by location shift, the misclassification error rate increased to 48.4% (IQR 46.9–52.0%) before normalization, and decreased back to 20.3% (IQR 18.8–21.9%) for median normalization, 23.4% (IQR 21.9–25.0%) for quantile normalization, and 23.4% (IQR 21.9–23.4%) for variance stabilizing normalization (Fig. 2B). Shifting the data for all markers up by a constant in half of the arrays did not change the order statistics in each of these arrays, so as expected the classification result remained the same once median or quantile normalization was applied.

When re-scaling handling effects differently across batches, misclassification errors increased to 26.6% (IQR 25.0–31.3%) before normalization and decreased to 20.3% (IQR 20.3–21.9%) for median normalization, 20.3% (IQR 18.8–25.0%) for quantile normalization, and 23.4% (IQR 21.9–23.4%) for variance stabilizing normalization (Fig. 2C).

These results suggest that the effects of normalization on prediction accuracy can depend on the level of signal-to-noise ratio and also the distributional characteristics of handling effects in the data.

### Relative performance of normalization methods depends on the classification algorithm

**Figure 3:**
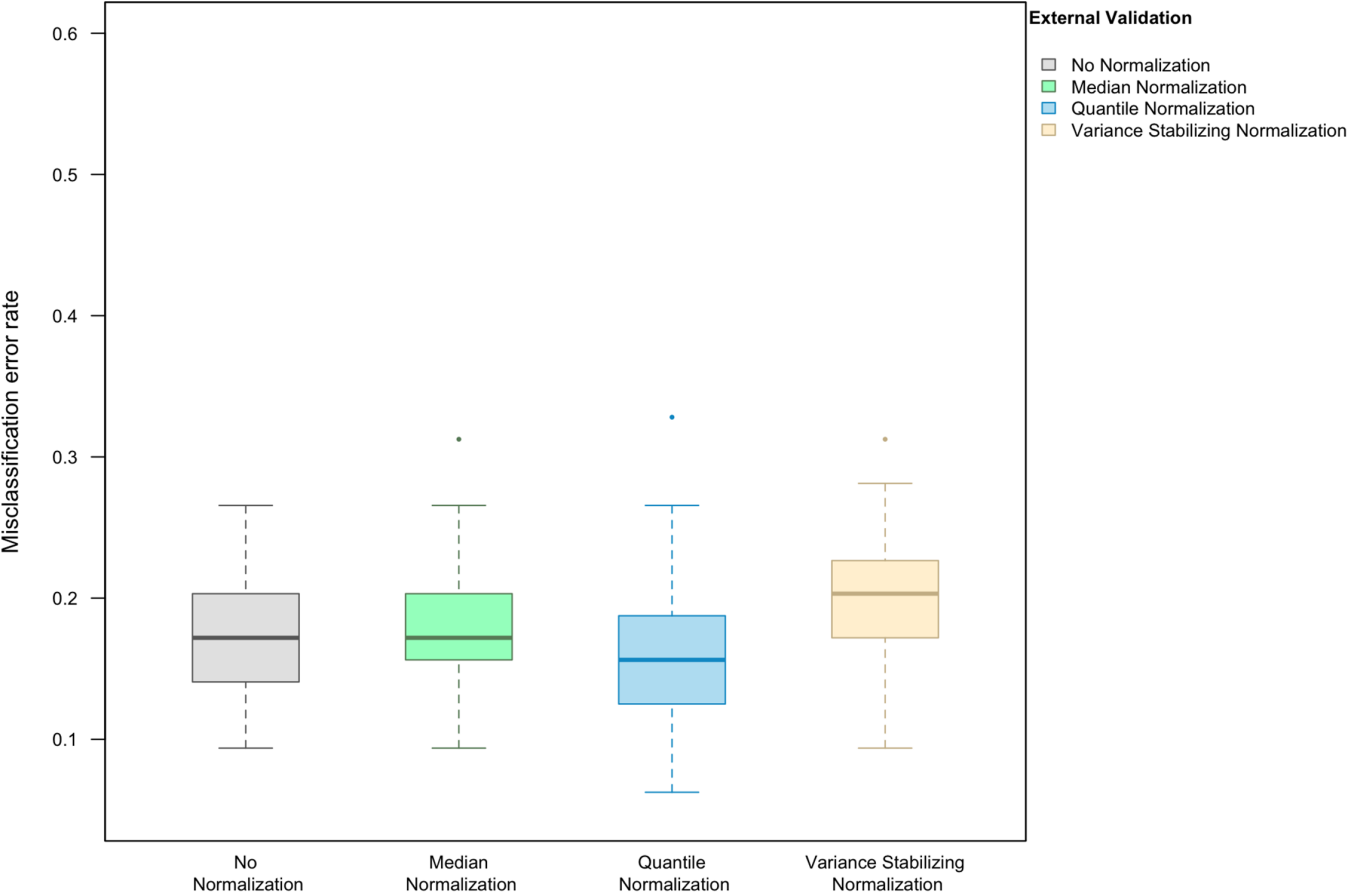
Boxplot of the misclassification error rate based on external validation when using the LASSO method for building a classifier. The simulated training data possessed no confounding handling effects. *x*-axis indicates the normalization method for the training data; *y*-axis indicates the misclassification error rate as a percentage.

**Figure 4:**
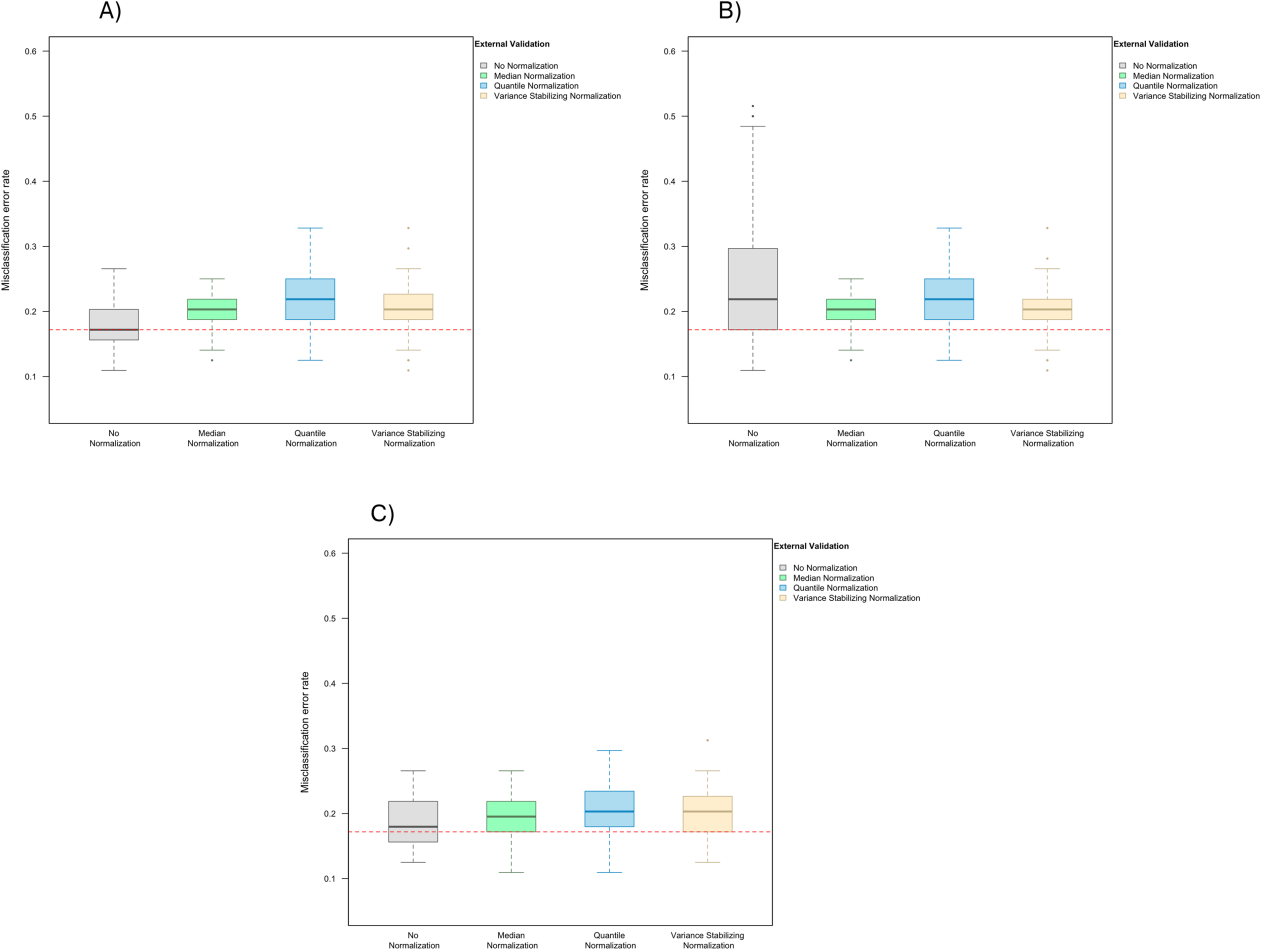
Boxplot of the misclassification error rates based on external validation when using the LASSO method for building a classifier. The simulated training data possessed confounding handling effects. *x*-axis indicates the normalization method for the training data; *y*-axis indicates the misclassification error rate as a percentage. (A–C) display the misclassification error when handling effects in the simulated training data were not amplified (A), amplified by location shift (B), and amplified by scale change (C). In (A–C) the horizontal red line indicates the level of classification error rate in the ideal case when the training data possessed no confounding handling effects and received no normalization.

When LASSO was used as the classification algorithm, data normalization did not provide an obvious benefit to classification accuracy: in the absence of handling effects, the misclassification error rate was 17.2% (IQR 14.1–20.3%) (Fig. 3); in the presence of handling effects, the rates were 17.2% (IQR 15.6–20.3%) before normalization, 20.3% (IQR 18.8–21.9%) after median normalization, 21.9% (IQR 18.8–25.0%) after quantile normalization, and 20.3% (IQR 18.8–22.3%) after variance stabilizing normalization (Fig. 4A).

When handling effects were amplified by location shift, normalization became beneficial for LASSO. We observed error rates of 21.9% (IQR 17.2–29.7%) before normalization, 20.3% (IQR 18.8–21.9%) after median normalization, 21.9% (IQR 18.8–25.0%) after quantile normalization, and 20.3% (IQR 18.8–21.9%) after variance stabilizing normalization (Fig. 4B). When handling effects were amplified by scale change, the signal-to-noise ratio did not decrease as dramatically as when they were amplified by location shift. As a result, normalization remained non-beneficial when LASSO was used: 18.0% (IQR 15.6–21.9%) before normalization, 19.5% (IQR 17.2–21.9%) after median normalization, 20.3% (IQR 18.4–23.4%) after quantile normalization, and 20.3% (IQR 17.2–22.3%) after variance stabilizing normalization (Fig. 4C).

In addition to using a matching normalization method for the test data, we again repeated the analysis comparing training set normalization methods when using only frozen quantile normalization for the test data, and observed similar results (Fig. S3).

### Over-optimistic estimation of classification accuracy for cross-validation

When compared with the error rate based on external validation, cross-validation was associated with under-estimation of the error rate regardless of the use of normalization for all three normalization methods examined in our study (Table S1; Fig. S4). The under-estimation is not inherent to cross-validation, as it was not associated with over-optimistic findings when used for the uniformly handled data, as reported in our previous work (Qin, Huang & Begg, 2016). Rather it is likely due to the over-compressed variability for the training data after normalization, leading to violation of the assumption of cross-validation that the training data is comparable with the test data.

## Discussion

We have shown through re-sampling-based simulations that normalization may improve the accuracy of molecular classification when the data possess confounding handling effects. The level of benefits and the relative performance between different normalization methods depended on the relative level of molecular signal, the distributional pattern of handling effects, and the statistical method used for classification. In our study, normalization led to a greater benefit for PAM than for LASSO, the mechanism of which will be explored in our future work. Comparing with its performance for the purpose of discovering differentially expressed biomarkers, median normalization is more on par with quantile normalization for the purpose of sample classification. We have made the data and code used for our study publicly available, so that other researchers interested in this topic can reproduce our study and further explore the use of additional normalization methods, classification methods, and simulation scenarios.

We have also shown that data normalization cannot restore the validity of cross-validation for estimating classification accuracy when the molecular data possess confounding handling effects. Our findings therefore reinforce the importance of generating quality data (that are free of confounding handling effects) through careful study design and controlled experimental process so that reproducible molecular classifiers can be derived and translated into clinical use.

## Conclusion

Normalization may be beneficial for improving the accuracy of molecular classification when the data possess confounding handling effects; however, it cannot mitigate the bias associated with cross-validation for estimating the classification accuracy.

## Supplemental Information

10.7717/peerj.4584/supp-1 Supplemental Information 1 An example array-to-sample-group assignment used in the study. X-axis indicates the array index and each strip represents an array. Strip color indicates the sample-group assignment: blue for endometrial samples, red for ovarian samples, and dark grey for not used in the simulations. Background shade color indicates the two technicians. Dashed lines separate arrays from different slides.

10.7717/peerj.4584/supp-2 Supplemental Information 2 Boxplot of the misclassification error rate based on external validation when using the PAM method for building a classifier. The simulated training data possess confounding handling effects. The test data were frozen quantile normalized regardless of the normalization method used for the training data. X-axis indicates the normalization method for the training data; y-axis indicates the misclassification error rate as a percentage.

10.7717/peerj.4584/supp-3 Supplemental Information 3 Boxplot of the misclassification error rate based on external validation when using the LASSO method for building a classifier. The simulated training data possess confounding handling effects. The test data were frozen quantile normalized regardless of the normalization method used for the training data. X-axis indicates the normalization method for the training data; y-axis indicates the misclassification error rate as a percentage.

10.7717/peerj.4584/supp-4 Supplemental Information 4 Boxplot of the misclassification error rate based on cross-validation in comparison with that based on external validation when using the PAM method (left column) or the LASSO method (right column) for building a classifier. The simulated training data possess confounding handling effects. The test data were frozen normalized to the training data using the matched normalization method. X-axis indicates the normalization method for the training data; y-axis indicates the misclassification error rate as a percentage. The three panels display the misclassification error when handling effects in the simulated training data were not amplified (A), amplified by location shift (B), and amplified by scale change (C).

10.7717/peerj.4584/supp-5 Supplemental Information 5 Summary statistics (median and inter-quartile range) of the misclassification error rate based on cross-validation in comparison with external validation when using either the PAM method (top) or the LASSO method (bottom) for building a classifier.

## Additional Information and Declarations

### Microarray Data Deposition

The following information was supplied regarding the deposition of microarray data: GSE109059: https://www.ncbi.nlm.nih.gov/geo/query/acc.cgi?acc=GSE109059.

### Data Availability

The following information was supplied regarding data availability: Github: https://github.com/LXQin/precisiondata; https://github.com/LXQin/precision.
