# Supplementary material for: Empirical evaluation of data normalization methods for molecular classification
Source: PeerJ. 2018 Apr 11;6:e4584. doi: 10.7717/peerj.4584 (PMC5899419; doi:10.7717/peerj.4584)
Supplement: Supplemental Information 1 — X-axis indicates the array index and each strip represents an array. Strip color indicates the sample-group assignment: blue for endometrial samples, red for ovarian samples, and dark grey for not used in the simulations. Background shade color indicates the two technicians. Dashed lines separate arrays from different slides. [file peerj-06-4584-s001.docx]

**Supplementary Figure 1. One of the possible array-to-sample-group assignments used in the study.**

X-axis indicates the array index and each strip represents an array. Strip color indicates the sample-group assignment: blue for endometrial samples, red for ovarian samples, and dark grey for not used in the simulations. Background shade color indicates the two technicians. Dashed lines separate arrays from different slides.

**
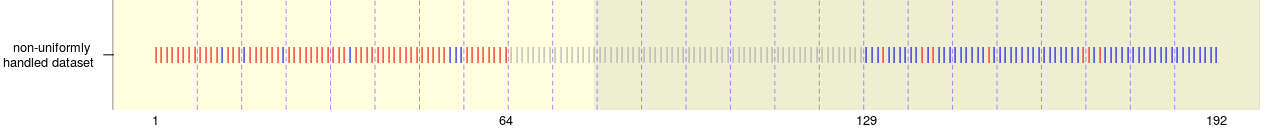
**
