# Supplementary material for: Empirical evaluation of data normalization methods for molecular classification
Source: PeerJ. 2018 Apr 11;6:e4584. doi: 10.7717/peerj.4584 (PMC5899419; doi:10.7717/peerj.4584)
Supplement: Supplemental Information 3 — The simulated training data possess confounding handling effects. The test data were frozen quantile normalized regardless of the normalization method used for the training data. X-axis indicates the normalization method for the training data; y-axis indicates the misclassification error rate as a percentage. [file peerj-06-4584-s003.docx]

**Supplementary Figure 3. Boxplot of the misclassification error rate based on external validation when using the LASSO method for building a classifier. The simulated training data possess confounding handling effects. The test data were frozen quantile normalized regardless of the normalization method used for the training data.**

X-axis indicates the normalization method for the training data; y-axis indicates the misclassification error rate as a percentage.

**
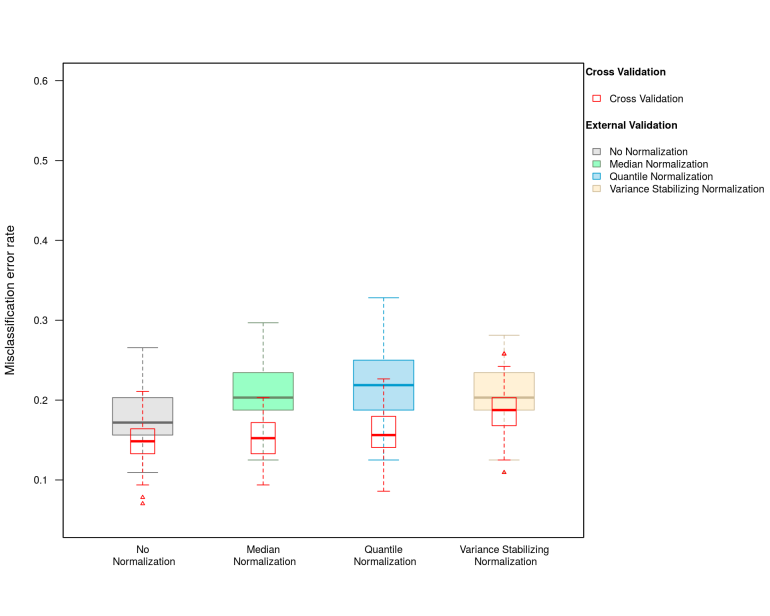
**
