# Supplementary material for: Empirical evaluation of data normalization methods for molecular classification
Source: PeerJ. 2018 Apr 11;6:e4584. doi: 10.7717/peerj.4584 (PMC5899419; doi:10.7717/peerj.4584)
Supplement: Supplemental Information 4 — The simulated training data possess confounding handling effects. The test data were frozen normalized to the training data using the matched normalization method. X-axis indicates the normalization method for the training data; y-axis indicates the misclassification error rate as a percentage. The three panels display the misclassification error when handling effects in the simulated training data were not amplified (A), amplified by location shift (B), and amplified by scale change (C). [file peerj-06-4584-s004.docx]

**Supplementary Figure 4. Boxplot of the misclassification error rate based on cross-validation in comparison with that based on external validation when using the PAM method (left column) or the LASSO method (right column) for building a classifier. The simulated training data possess confounding handling effects. The test data were frozen normalized to the training data using the matched normalization method.**

X-axis indicates the normalization method for the training data; y-axis indicates the misclassification error rate as a percentage. The three panels display the misclassification error when handling effects in the simulated training data were not amplified (A), amplified by location shift (B), and amplified by scale change (C).

**A.**

**
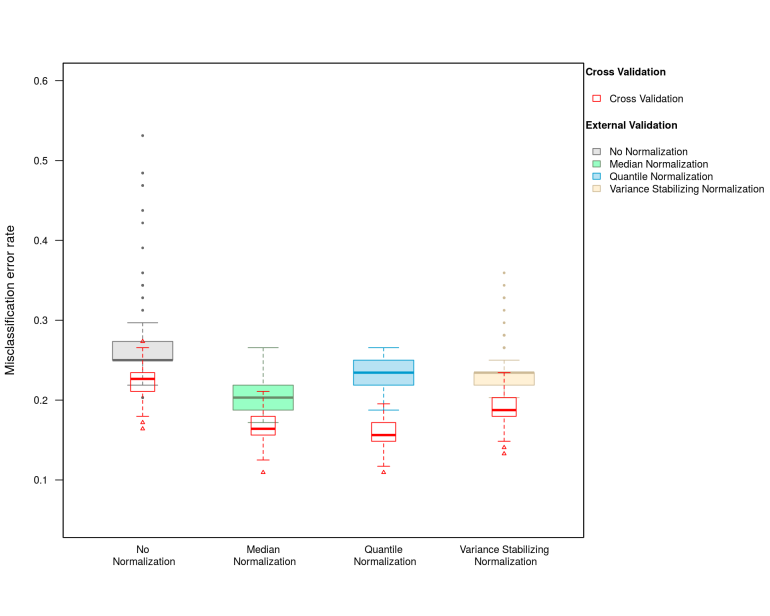

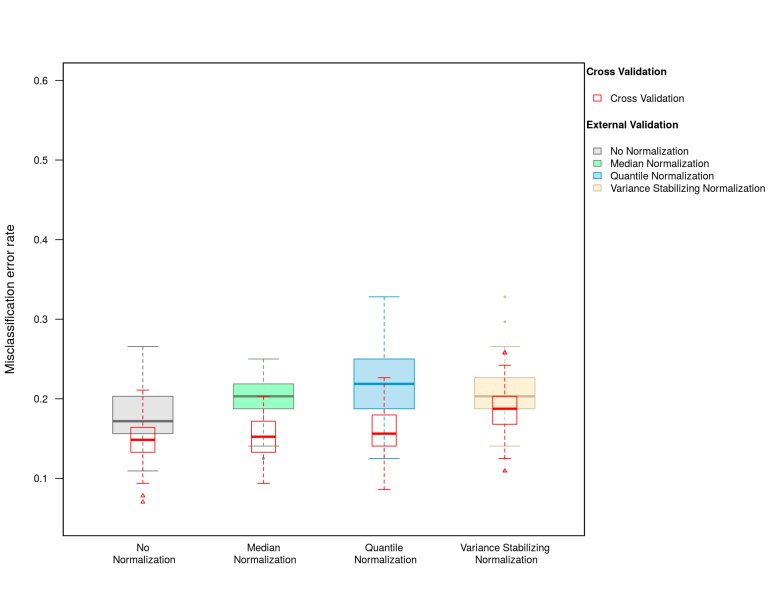
**

**B.**


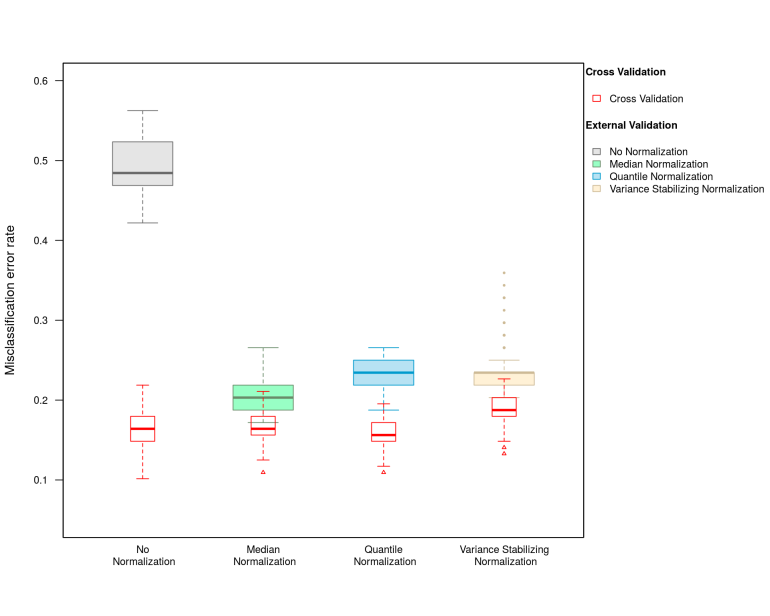

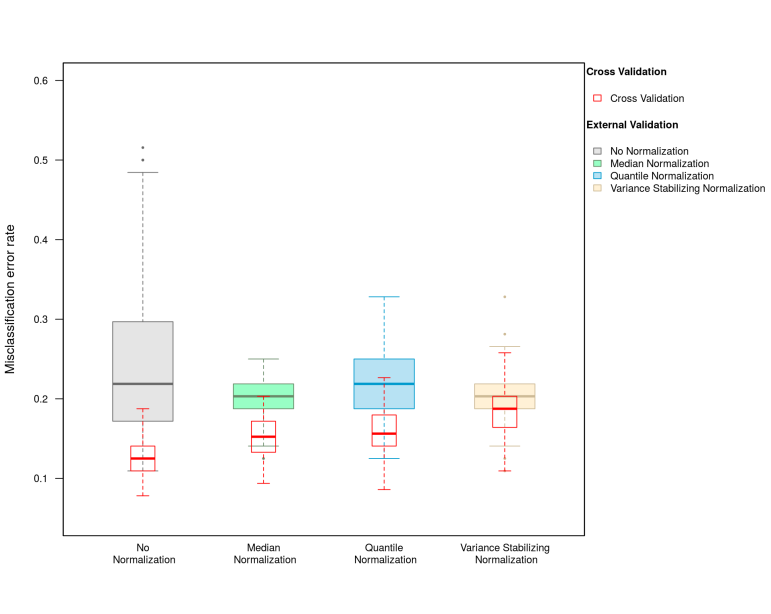


**C.**

**
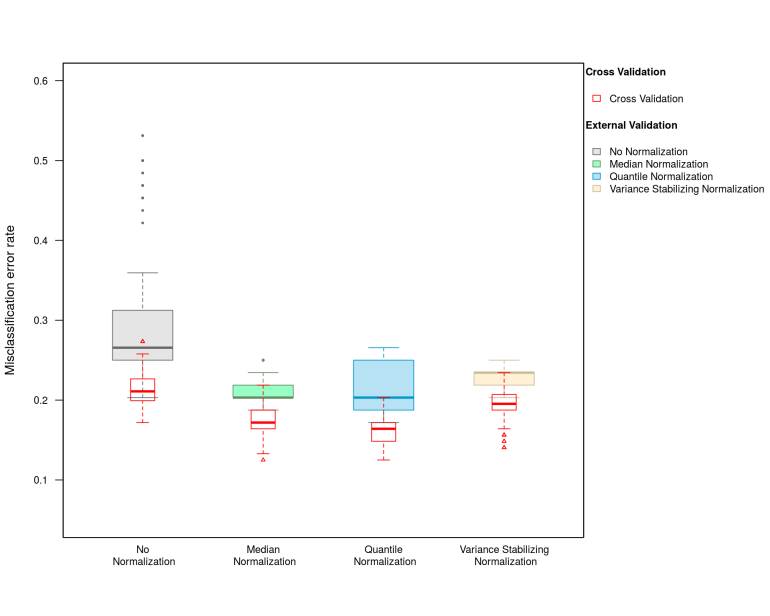

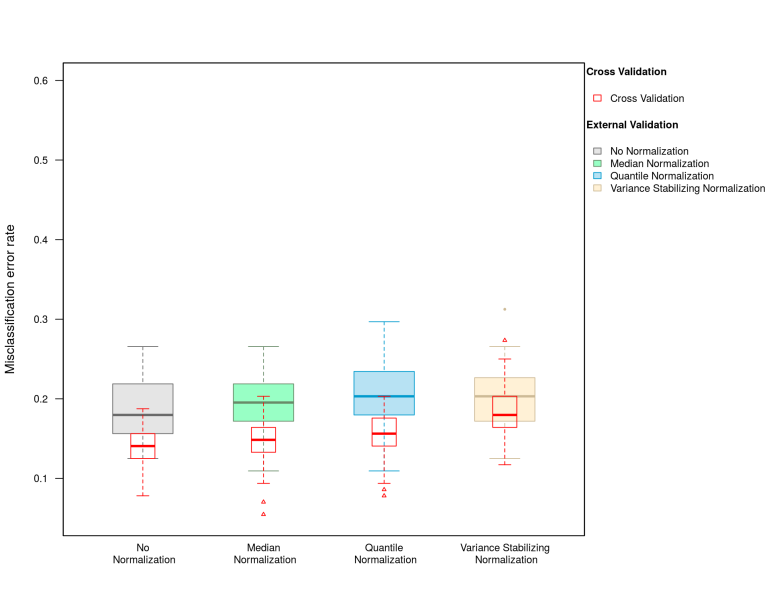
**
