# Supplementary material for: Empirical evaluation of data normalization methods for molecular classification
Source: PeerJ. 2018 Apr 11;6:e4584. doi: 10.7717/peerj.4584 (PMC5899419; doi:10.7717/peerj.4584)
Supplement: Supplemental Information 5 [file peerj-06-4584-s005.docx]

Supplementary Table 1. Summary statistics (median and inter-quartile range) of the misclassification error rate based on cross-validation in comparison with external validation when using either the PAM method (top) or the LASSO method (bottom) for building a classifier.

| PAM | | | | | | | |
| --- | --- | --- | --- | --- | --- | --- | --- |
| No  Normalization | | **Median**  **Normalization** | | **Quantile**  **Normalization** | | **Variance Stabilizing Normalization** | |
| *External validation* | *Cross-validation* | *External validation* | *Cross-validation* | *External validation* | *Cross-validation* | *External validation* | *Cross-validation* |
| 25.0% | **22.7%** | **20.3%** | **16.4%** | **23.4%** | **15.6%** | **23.4%** | **18.8%** |
| 25.0%–27.0% | 21.1%–23.4% | 18.8%–21.9% | 15.6%–18.0% | 21.9%–25.0% | 14.8%–17.2% | 21.9%–23.4% | 18.0%–20.3% |

| LASSO | | | | | | | |
| --- | --- | --- | --- | --- | --- | --- | --- |
| No  Normalization | | **Median**  **Normalization** | | **Quantile**  **Normalization** | | **Variance Stabilizing Normalization** | |
| *External validation* | *Cross-validation* | *External validation* | *Cross-validation* | *External validation* | *Cross-validation* | *External validation* | *Cross-validation* |
| 17.2% | **14.8%** | **20.3%** | **15.2%** | **21.9%** | **15.6%** | **20.3%** | **18.8%** |
| 15.6%–20.3% | 13.3%–16.4% | 18.8%–21.9% | 13.3%–17.2% | 18.8%–25.0% | 14.1%–18.0% | 18.8%–22.3% | 17.0%–20.3% |
